# Relationship between phonological working memory, metacognitive skills and reading comprehension in children with learning disabilities

**DOI:** 10.1590/1678-7757-2017-0414

**Published:** 2018-07-19

**Authors:** Ana Paola Nicolielo-Carrilho, Patrícia Abreu Pinheiro Crenitte, Simone Aparecida Lopes-Herrera, Simone Rocha de Vasconcellos Hage

**Affiliations:** 1 Universidade de São Paulo, Faculdade de Odontologia de Bauru, Departamento de Fonoaudiologia, Bauru, São Paulo, Brasil.

**Keywords:** Learning, Short-term memory, Reading, Comprehension, Child language

## Abstract

Reading requires the activation of several cognitive processes, some of which are basic, e.g. recognizing letters and words, whereas others are complex, such as working memory and ability to think about one's own learning strategies. One condition for fulfilling a complex cognitive task, such as understanding a text, is the ability to maintain and process information, which depends on working memory.

**Objective:**

To analyze the ability of using metacognitive strategies for reading, the phonological working memory of school children with learning disabilities, and also determine if there is relation between these skills and reading comprehension.

**Method:**

The sample consisted of 30 school-age children and teenagers of both genders, aged 8 to 12 years, who were enrolled in primary school. They were divided in two groups, experimental (EG) and control (CG). All children were subjected to evaluation of reading comprehension, phonological working memory, and use of metacognitive skills for reading. The results were compared between groups through the Mann-Whitney test, and correlation between variables was analyzed through Spearman correlation test.

**Result:**

Statistical comparison between EG and CG showed statistically significant difference. Positive and effective correlation was observed between reading comprehension, phonological working memory and metacognitive tests.

**Conclusion:**

children with learning disabilities presented deficits in phonological working memory and use of metacognitive strategies. The positive and effective correlation between the abilities analyzed suggests that failure in the phonological working memory and use of metacognitive strategies interfere with reading comprehension.

## Introduction

Reading requires the activation of several cognitive processes, such as recognizing letters and words, working memory, and the ability to think about one's own learning strategies.

One condition required to fulfill a complex cognitive task, such as understanding a text, is the ability to maintain and process information, which depends on working memory. One component of working memory is the phonological loop, whose function is temporarily storing linguistic information.^2^ This phonological component of the working memory plays an important role in learning how to read, oral language comprehension, vocabulary acquisition, and reading comprehension.^5^ ^,^ ^12^

The understanding of people about their own cognitive processing is called metacognition.^8^ Metacognitive abilities correspond to a subsystem of control within the cognitive system, whose goal is to monitor, plan and regulate its processes, a high-level processing played by the executive function.^10^ The role of metacognitive capacity on the learning processes is undisputable, and metacognitive learning strategies are procedures used by students to plan, monitor, and regulate their own thinking in order to acquire certain knowledge.^30^ These strategies have been related with reading comprehension, as their use allows the reader to have more chance to understand texts compared to individuals who do not use them.^13^ ^,^ ^15^ Good readers not only decode what they are reading, but also review and question the meanings of reading and try to determine the meaning of familiar words and concepts, aside from solving difficulties as they arise.

Reading difficulties presented by students may be caused by brain disorders related to the ability of learning to read and write, as observed in learning disabilities (LD). Learning disabilities is a broad term, indicating that a child's achievement is considerably below the expected threshold. The term does not include disorders primarily caused by intellectual disabilities, emotional, visual or hearing disorders. Even though these children present average or above average level of intelligence, they have difficulties in acquiring basic academic skills, such as correct spelling, fluent reading, written expression and mathematical calculations, according to the National Joint Committee for Learning Disabilities.^16^

The hypothesis is that difficulties in text comprehension by children with learning disabilities may be explained by failure in the phonological component of working memory and in the metacognitive ability to plan, monitor and regulate their own thinking for understanding a text.^24^

The purpose of this study was to analyze the ability of using metacognitive strategies for reading, the phonological working memory in school children with learning disabilities, as well as to determine if there is relation between these skills and reading comprehension.

## Material and methods

This quantitative experimental study was approved by the Institutional Review Board of the University of São Paulo. The sample was composed of 30 school children and teenagers of both genders, aged 8 to 12 years, who were enrolled in primary school. The individuals were divided in two groups, namely experimental (EG) and control (CG).

The experimental group included 15 children and teenagers diagnosed with learning disabilities by an interdisciplinary team from the Diagnostic Center of Language and Learning Alterations of the University of São Paulo. In this center, all 15 subjects from the experimental group underwent neurological, neuropsychological, language and audiological assessments. The study also comprised an interview with the pedagogical coordinator or teacher from the school where the individuals studied to verify the educational proposals offered by the Institution. The criteria for diagnosis of learning disabilities were those cited by the National Joint Committee on Learning Disabilities.^16^

The control group was composed of 15 school age children or teenagers with no language and learning impairments. As inclusion criteria, the students should be regularly attending primary school, not have flunked any grade, and not present complaints of learning and/or history of disorders in language and hearing development. They should also present average or higher performance in writing (word dictation), arithmetic, and reading of isolated words, according to their school grade in the School Achievement Test.^27^

All children in the experimental and control groups were evaluated as to their ability of reading comprehension, phonological working memory and utilization of metacognitive strategies for reading. No children in the sample had visual complaints that might impair the accomplishment of the tests.

Reading comprehension was evaluated using the subtest – text comprehension – of the “Evaluation of Processes of Reading” (Prolec)^6^ and the fixed-ratio Cloze test. The Prolec subtest of text comprehension is composed of four texts, two being of narrative and two of expositive nature, which should be read by the child and, for each of them, the child should respond to four questions, two being literal and two inferential.

A text with approximately 250 words was selected for application of the fixed-ratio Cloze Procedure,^28^ according to the child's educational level. The Cloze test comprises selection of a text, in which the fifth word is systematically omitted after the second sentence. The task requires the reader to process the information from the text, select a word to better fill the gap and check if the word is adequate for use. These words may be inferred from the text itself (intratextual inference), or from previous knowledge of the reader (extratextual inference). The correct answers were added, and the total score was converted to a percentage of correct answers. The child's performance was scored as described by Bormuth^3^ (1992), as follows: independent (excellent, 90 to 100%; and good, 75 to 89%), intermediate with need of additional aid (instructional, 58 to 74%; and with difficulties, 44 to 57%), and deficient (bad reader, 30 to 43%; and terrible reader, below 30%).

The phonological working memory was assessed by the Nonword Repetition Test^11^, which evaluates the number of items the individual is able to retain and retrieve in memory, immediately after the oral presentation of a list of nonwords. An appropriate response is considered if the repetition is identical to that presented by the examiner, who is allowed to repeat every word only once.

The metacognitive strategies used for reading were evaluated by the Reading Strategies Scale – Fundamental Level I,^7^ which aims to evaluate the type of metacognitive strategies employed by the children before, during and after the reading of child's literary texts. The scale is composed of 13 Likert statements with three possibilities of responses (never: 0 point; sometimes: 1 point; always: 2 points). The first five statements encompass metacognitive strategies for reading support (factor 1), the following five are related with strategies used to solve comprehension problems (factor 2), and the three last statements group strategies used for overall analysis of the text (factor 3). The statements are also analyzed according to the moment when reading is performed: before (3 questions), during (6 questions) and after (3 questions). The sum of scores may be performed by factor (1, 2 and 3), by moment in relation to reading (before, during and after), and by the sum of all points.

**Figure 1:**
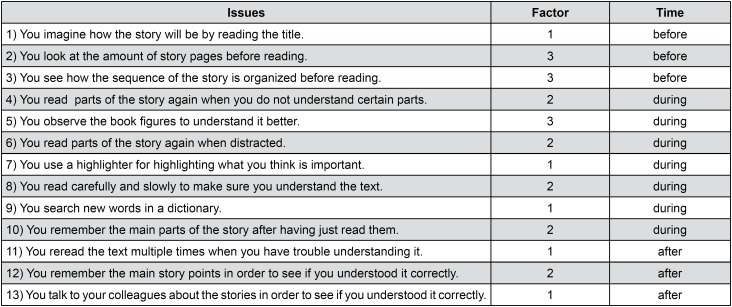
Strategy range of reading issues and their classification according to factor and time

Figure 1 presents the questions of the Reading Strategies Scale, their factor and moment.

Direct scores of each procedure applied were used. The results were analyzed according to each specific procedure and tabulated on Microsoft Excel^®^ worksheets. Comparison between groups was performed by the Mann-Whitney test. The correlation between variables of interest (abilities analyzed and reading comprehension) was assessed by the Spearman correlation analysis. All tests were applied at a significance level of 5% (0.050).

## Results

The sample consisted of 30 children, the majority of whom were male (24 boys and 6 girls), who were equally divided in experimental and control groups, with mean age of 9.8 years, enrolled in the 3^rd^ to 6^th^ grades of primary public schools.

Table 1 presents the means, medians, standard deviations, and a comparison between EG and CG.

**Table 1:** Performance comparison between experimental and control groups in the tests

Variable	Group	N	Mean	Standard Deviation	Min	Max	25 percent	50 percent	75 percent	Sig. (p)
Nonword Repetition Test	EG	15	55.67	12.74	35.00	78.00	45.00	58.00	64.00	<0.001
	CG	15	77.40	3.02	72.00	80.00	76.00	79.00	80.00	
Reading strategy scale – Total score	EG	15	11.87	5.62	2.00	22.00	9.00	11.00	16.00	0.015
	CG	15	16.87	4.29	11.00	24.00	13.00	19.00	20.00	
Support strategies factor 1	EG	15	4.13	2.64	0.00	10.00	2.00	4.00	6.00	0.091
	CG	15	5.67	1.99	3.00	10.00	4.00	6.00	6.00	
Solution strategies factor 2	EG	15	3.93	2.69	0.00	8.00	2.00	3.00	7.00	0.005
	CG	15	7.00	2.45	2.00	10.00	6.00	8.00	9.00	
Global strategies factor 3	EG	15	3.80	1.66	1.00	6.00	2.00	4.00	5.00	0.703
	CG	15	4.20	1.94	2.00	9.00	3.00	4.00	5.00	
Strategies before reading	EG	15	2.93	1.62	0.00	6.00	2.00	3.00	4.00	0.278
	CG	15	3.53	1.36	2.00	6.00	2.00	3.00	4.00	
Strategies while reading	EG	15	6.00	3.68	0.00	12.00	3.00	5.00	9.00	0.127
	CG	15	7.73	2.22	5.00	12.00	5.00	8.00	9.00	
Strategies after reading	EG	15	2.60	1.35	0.00	5.00	2.00	3.00	3.00	<0.001
	CG	15	5.60	1.84	2.00	8.00	4.00	6.00	7.00	
Subtest Reading Comprehension – PROLEC	EG	15	7.60	5.05	0.00	14.00	0.00	8.00	11.00	<0.001
	CG	15	14.40	1.50	12.00	16.00	14.00	14.00	16.00	
Inferential questions	EG	15	3.07	2.22	0.00	6.00	0.00	3.00	5.00	<0.001
	CG	15	7.07	1.03	5.00	8.00	7.00	7.00	8.00	
Literal questions	EG	15	4.53	2.90	0.00	8.00	0.00	5.00	7.00	<0.001
	CG	15	7.33	0.72	6.00	8.00	7.00	7.00	8.00	
Cloze Test	EG	15	7.87	6.90	0.00	21.00	0.00	8.00	14.00	<0.001
	CG	15	28.80	2.37	25.00	32.00	27.00	29.00	31.00	

Regarding statistical analysis and comparison between groups, as shown in the table presented above, statistical comparison showed statistically significant difference (<0.001) in phonological working memory, two tests of reading comprehension. Children in the EG also presented worse performance in the usage of metacognitive strategies in general compared to those without difficulties (p<0.015), but not for all questions analyzed. Even though the EG presented worse performance in all of them, significant difference was observed in them regarding reading support and resolution of comprehension problems (factors 1 and 2).

Table 2 presents the analysis with p and r values for the EG, considering the skills analyzed.

**Table 2:** Spearman correlation analysis between the experimental groups (EG) evaluated skills and reading comprehension tests

		Text comprehension PROLEC			
Variable	Statistics	Total Score	Inferential Questions	Literal Questions	Cloze Test
Nonword Repetition Test	correlation coefficient (r) Sig. (p)	0.655 0.008	0.673 0.006	0.643 0.010	0.725 0.002
Reading strategy scale – Total score	correlation coefficient (r) Sig. (p)	0.533 0.041	0.521 0.047	0.583 0.022	0.361 0.186
Support strategies factor 1	correlation coefficient (r) Sig. (p)	0.463	0.479	0.513	0.330
		0.082	0.071	0.051	0.229
Solution strategies factor 2	correlation coefficient (r) Sig. (p)	0.636 0.011	0.593 0.020	0.675 0.006	0.479 0.071
Global strategies factor 3	correlation coefficient (r) Sig. (p)	0.149 0.597	0.107 0.705	0.237 0.395	0.195 0.487
Strategies before reading	correlation coefficient (r) Sig. (p)	0.254 0.361	0.186 0.506	0.380 0.163	0.267 0.336
Strategies while reading	correlation coefficient (r) Sig. (p)	0.463 0.082	0.471 0.076	0.522 0.046	0.370 0.175
Strategies after reading	correlation coefficient (r) Sig. (p)	0.451 0.092	0.445 0.097	0.402 0.138	0.258 0.353

As shown, correlation between phonological working memory and reading comprehension was positive in the group of children with learning disabilities. Phonological working memory presented parallel behavior with reading comprehension, i.e. the best the performance in working memory, the best was the performance in reading comprehension and vice versa. Considering *p*-value (smaller than or equal to 0.050), the relationship between phonological working memory and reading comprehension was effective for both procedures, Prolec text comprehension and Cloze test.

The correlation between the overall performance in the reading strategies scale and the reading comprehension tests was also positive, namely, the better the performance in the reading strategies scale, the better the performance in reading comprehension. Statistically significant correlations were observed between the reading strategies scale and the Prolec text comprehension test.

**Figure 2:**
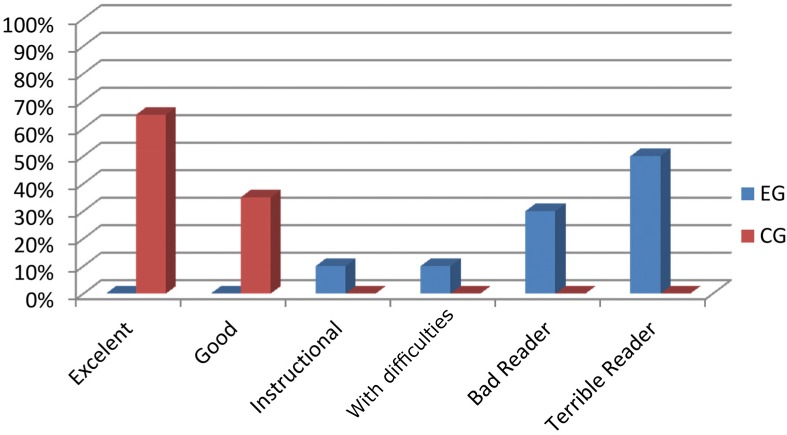
Percentage of ratings occurrence of Cloze Test in the experimental and control groups

Figure 2 displays the percentage of occurrence of each rating of the Cloze test in the sample studied.

## Discussion

Concerning the occurrence of learning difficulties, in Brazil and other developing countries, it is estimated that 40% to 42% of students in initial grades present these difficulties; among these, 4% to 6% present neurobiological disorders,^25^ as observed with learning disabilities. In individuals with learning disabilities, problems for reading and writing may manifest more clearly during formal educational, even though its manifestations occur before the 1^st^ grade, especially at the first stages of primary school. Regarding the prevalence of this disorder, higher occurrence is observed in males,^23^ which was also observed in this study.

Children with learning disabilities present inaccurate and slow reading, and this effort spent to the basic activity of decoding precludes them from establishing connections between the several elements of the text. Several abilities constitute the basis of the learning process, including the phonological working memory.

Working memory is composed of several cognitive processes, which combine both storage and processing of information. Baddeley^2^ (2006) described the components of this system, especially focusing on a component called phonological loop, whose function is the temporal storage of limited linguistic information. This phonological component plays a role in the understanding of oral language, vocabulary acquisition, and reading comprehension.^5^ Deficits in the phonological working memory may be frequently observed in school age children with learning disorders,^29^ as confirmed by this study.

Learning and memory are strictly related processes, thus it may be assumed that memory disturbances can cause damages in learning.^1^ ^,^ ^5^ Specifically about reading skills, the working memory is considered a well-established predictor of individual variation in the reading comprehension of children and adults.^19^

Memory plays an important role, considering that understanding a text requires remembering what was read. Reading textual input activates the related knowledge stored in the long-term memory, bringing them to the working memory, which allows for the temporary storage of information presented in the text and previous information recalled from the long-term memory, so as the reader may construct relationships between them, i.e. the meaning of the text.^15^ ^,^ ^19^ Thus, failures in working memory would impair the accomplishment and completion of the entire process.

Researchers have been increasingly interested in the training of working memory and to which extent such training might strengthen the verbal comprehension of young children at risk of learning difficulties.^21^ Working memory plays an important role in the academic performance of children, as several academic tasks involve multiple sequences of tasks that should be remembered in a short period of time. When reading a text, children should remember previously learnt information, adding the information received to their knowledge inventory as they advance in reading.

Investigations on reading comprehension have widened knowledge on this topic, demonstrating the contribution of several abilities for its development, including the ability of reading comprehension monitoring.^9^ Learning disabilities also imply failures in the processing of auditory, linguistic and cognitive information, which negatively influence the action of metacognitive mechanisms to plan, monitor and regulate own thinking in order to acquire certain knowledge,^26^ which was confirmed by the results of this study. The EG exhibited difficulties involving reading support and resolution of comprehension problems such as rereading parts of the story when the child did not understand or was distracted, remembering the main parts of the story soon after finishing reading, or remembering the main parts of the story to check if he or she understood.

When using metacognitive strategies for reading, readers have more chances to understand the text compared to individuals that do not use them.^13^ During reading, readers may employ different reading strategies, and their choices of certain strategies in that particular context will directly influence their reading comprehension.^17^ In a certain context, even without notice, the reader chooses one or more strategies that are pertinent to that situation, while another strategy may be chosen in another context. Knowing such strategies would enhance the pertinent selection and utilization of them by the reader as a facilitator of the comprehension process. Children without learning difficulties develop individual strategies that enhance text comprehension by their own. Conversely, children with learning disabilities require special support, either because they do not develop them or because they use strategies that are ineffective.

Metacognitive activities may enhance the reading comprehension, improving the chances of text comprehension by school-age children compared to those who do not perform these activities or perform them inefficiently.^4^ ^,^ ^13^ ^,^ ^15^ ^,^ ^22^ Demonstrating the relationship between the ability of using reading strategies affects the therapeutic process of children with LD. Metacognitive abilities should be further analyzed, both by speech-language pathologists and by pedagogues in the classroom, since their use may aid in the evolution of text comprehension in children with learning difficulties.^18^

Even though difficulties in learning comprehension are expected in children with LD, assessing the degree of this difficulty and if there is difference in performance when reading comprehension depends on inferences brings about implications for therapeutic planning.

In the Cloze test, children in the EG showed poor reading level or in need of additional aid. A bad performance in the Cloze test indicates that the reader, even with aid, presents difficulty in using contextual clues or activating previous knowledge in order to understand the text.^14^ Accomplishment of the Cloze activity assumes that reading comprehension results from underlying cognitive processes that include not only the decoding of symbols and perception of clues inside the text, but also the retrieval of previous knowledge from within the memory, an ability that may be impaired in children with learning disabilities.

In the Prolec text comprehension subtest, children with learning disabilities presented worse performance in inferential questions compared to literal questions. Conversely, the performance of the control group was similar for both question types (Table 1). Children with worse performance in question comprehension, in particular inferential questions, presented lower scores in the reading strategies scale, indicating that the ability to inference involves metacognitive strategies. Inferences are beyond just memory usage and imply text interpretation. The inferential process assures the organization of senses designed by the reader in its relationship with the text, only made possible based on the relationship between parts of the text as well as between them and the context. Qualified readers present better capability of making inferences, which allows them to relate ideas of the text, facilitating the comprehension.^20^ Inferences are fundamental for comprehension, because they allow the reader to complete the information that is missing in the text, leading the reader to a global and effective comprehension of the text.

## Conclusion

Children with learning disabilities presented deficits in phonological working memory and use of metacognitive strategies. The positive and effective correlation between the abilities analyzed suggests that failure in phonological working memory and use of metacognitive strategies interfere with the reading comprehension.

The occurrence of deficits in reading comprehension in individuals diagnosed with learning disabilities is widely discussed in the literature and therefore warrants special attention. Developing training programs targeted to the phonological memory, as already observed for phonological awareness, would positively influence the development of skillful readers. Similarly, the exploitation of metacognitive abilities, both in the clinical and educational environments, would certainly be beneficial for children presenting some type of learning disorder or difficulty. Many children will learn to read and write without any difficulty, yet others will require help to achieve success in the same activity.

## References

[1] Arrington CN, Kulesz PA, Francis DJ, Fletcher JM, Barnes MA (2014). The contribution of attentional control and working memory to reading comprehension and decoding. Sci Stud Read..

[2] Baddeley AD, Pickering S (2006). Working memory: an overview. Working memory and education.

[3] Bormuth JR (1992). Cloze test readability: criterion reference scores. J Educ Meas..

[4] Bruce ME, Robinson GL (2000). Effectiveness of metacognitive reading program for poor readers. International Educ Res..

[5] Cain K, Oakhill J, Bryant P (2004). Children's reading comprehension ability: concurrent prediction by working memory, verbal ability, and component skills. J Educ Psychol..

[6] Capellini SA, Oliveira AM, Cuetos F (2010). PROLEC: Provas de avaliação dos processos de leitura.

[7] Carvalho MR (2006). Estratégias metacognitivas de leitura utilizadas de 2^a^ a 5^a^ série do ensino fundamental.

[8] Chevalier TM, Parrila R, Ritchie KC, Deacon SH (2017). The role of metacognitive reading strategies, metacognitive study and learning strategies, and behavioral study and learning strategies in predicting academic success in students with and without a history of reading difficulties. J Learn Disabil..

[9] Coelho CL, Correa J (2017). Compreensão de leitura: habilidades cognitivas e tipos de texto. Psico (Porto Alegre).

[10] Destan N, Hembacher E, Gheti S, Roebers CM (2014). Early metacognitive abilities: the interplay of monitoring and control process in 5- to 7-year old children. J Exp Child Psychol.

[11] Hage SR, Grivol MA (2009). Reference values of nonword repetition test for Brazilian Portuguese-speaking children. J Appl Oral Sci.

[12] Hoff E, Core C, Bridges K (2008). Non-word repetition assesses phonological memory and is related to vocabulary development in 20- to 24-month- olds. J Child Lang.

[13] Lau K, Chan DW (2003). Reading strategy use and motivation among Chinese good and poor readers in Hong Kong. J Res Read.

[14] Maki RH, Schields M, Wheeler AE, Zacchini TL (2005). Individual differences in absolute and relative meta-comprehension accuracy. J Educ Psychol.

[15] Mokhtari K, Reichard CA (2002). Assessing student's metacognitive awareness of reading strategies. J Educ Psychol.

[16] National Joint Committee for Learning Disabilities (1994). Issues in learning disabilities: assessment and diagnosis [Internet].

[17] Nevo E, Bar-Kochva I (2015). The relations between early working memory abilities and later developing reading skills: a longitudinal study from kindergarten to fifth grade. Mind Brain Educ.

[18] Nicolielo-Carrilho AP, Hage SR (2017). Metacognitive reading strategies of children with learning disabilities. Codas.

[19] Nouwens S, Groen MA, Verhoeven L (2017). How working memory relates to children's reading comprehension: the importance of domain-specificity in storage and processing. Read Writ.

[20] Oakhill J (1984). Inferencial and memory skills in children's comprehension of stories. Br J Educ Psychol.

[21] Peng P, Fuchs DA (2017). A randomized control trial of working memory training with and without strategy instruction: effects on young children's working memory and comprehension. J Learning Disabil.

[22] Pressley M, Gaskin IW (2006). Metacognitively competent reading comprehension is constructively responsive reading: how can such reading developed is student?. Metacognition Learn.

[23] Quinn JM, Wagner RK (2015). Gender differences in reading impairment and in the identification of impairment readers. Results from a large-scale study of at-risk readers. J Learn Disabil.

[24] Schroeder PJ (2014). The effects of age on processing and storage in working memory span tasks and reading comprehension. Exp Aging Res.

[25] Silva C, Capellini SA (2013). Desempenho de escolares com e sem transtorno de aprendizagem em leitura, escrita, consciência fonológica, velocidade de processamento e memória de trabalho fonológica. Rev Psicopedag.

[26] Silver CH, Ruff RM, Iverson GL, Barth JT, Broshek DK, Bush SS (2008). Learning disabilities: the need for neuropsycological evaluation. Arch Clin Neuropsychol.

[27] Stein LM (1994). TDE – Teste de Desempenho Escolar: manual para aplicação e interpretação.

[28] Taylor WL (1953). “Cloze procedure”: a new tool for measuring readability. Journalism Quarterly.

[29] Wang S, Gathercole SE (2013). Working memory deficits in children with reading difficulties: Memory span and dual task coordination. J Exp Child Psychol.

[30] Weinstein CE, Acee TW, Jung J (2011). Self regulation and learning strategies. New Dir Teach Learn.

